# Inhibition of Fatty Acid Oxidation Promotes Macrophage Control of Mycobacterium tuberculosis

**DOI:** 10.1128/mBio.01139-20

**Published:** 2020-07-07

**Authors:** Pallavi Chandra, Li He, Matthew Zimmerman, Guozhe Yang, Stefan Köster, Mireille Ouimet, Han Wang, Kathyrn J. Moore, Véronique Dartois, Joel D. Schilling, Jennifer A. Philips

**Affiliations:** a Division of Infectious Diseases, Department of Medicine, Washington University School of Medicine, St. Louis, Missouri, USA; b Department of Molecular Microbiology, Washington University School of Medicine, St. Louis, Missouri, USA; c Department of Pathology and Immunology, Washington University School of Medicine, St. Louis, Missouri, USA; d Cardiovascular Division, Department of Medicine; Washington University School of Medicine, St. Louis, Missouri, USA; e Center for Discovery and Innovation, Hackensack Meridian Health, Nutley, New Jersey, USA; f Division of Infectious Diseases and Immunology, Department of Medicine, New York University Medical Center, New York, New York, USA; g Marc and Ruti Bell Vascular Biology and Disease Program, Leon H. Charney Division of Cardiology, Department of Medicine, New York University Medical Center, New York, New York, USA; Yale University School of Medicine

**Keywords:** *Mycobacterium tuberculosis*, NADPH oxidase, fatty acid oxidation, innate immunity, macrophages, mitochondrial metabolism

## Abstract

Macrophage activation involves metabolic reprogramming to support antimicrobial cellular functions. How these metabolic shifts influence the outcome of infection by intracellular pathogens remains incompletely understood. Mycobacterium tuberculosis (Mtb) modulates host metabolic pathways and utilizes host nutrients, including cholesterol and fatty acids, to survive within macrophages. We found that intracellular growth of Mtb depends on host fatty acid catabolism: when host fatty acid β-oxidation (FAO) was blocked chemically with trimetazidine, a compound in clinical use, or genetically by deletion of the mitochondrial fatty acid transporter carnitine palmitoyltransferase 2 (CPT2), Mtb failed to grow in macrophages, and its growth was attenuated in mice. Mechanistic studies support a model in which inhibition of FAO generates mitochondrial reactive oxygen species, which enhance macrophage NADPH oxidase and xenophagy activity to better control Mtb infection. Thus, FAO inhibition promotes key antimicrobial functions of macrophages and overcomes immune evasion mechanisms of Mtb.

## Abstract (precis)

Mycobacterium tuberculosis (Mtb) is the leading infectious disease killer worldwide. We discovered that intracellular Mtb fails to grow in macrophages in which fatty acid β-oxidation (FAO) is blocked. Macrophages treated with FAO inhibitors rapidly generate a burst of mitochondria-derived reactive oxygen species, which promotes NADPH oxidase recruitment and autophagy to limit the growth of Mtb. Furthermore, we demonstrate the ability of trimetazidine to reduce pathogen burden in mice infected with Mtb. These studies will add to the knowledge of how host metabolism modulates Mtb infection outcomes.

## INTRODUCTION

Macrophages are at the forefront of innate immune defense and are required for microbial killing, tissue homeostasis and repair, inflammation, and development. A wealth of scientific studies describes how these cells recognize pathogen-associated molecular patterns (PAMPs), which activate downstream signaling cascades to drive proinflammatory responses and resolve infection. Macrophage activation involves profound metabolic reprogramming to support specific phenotypic functions. Exposure of macrophages to stimuli such as gamma interferon (IFN-γ) and lipopolysaccharide (LPS) induces an inflammatory phenotype characterized by enhanced glycolytic metabolism and impaired oxidative phosphorylation (OXPHOS), similar to the Warburg effect described for cancer cells. Glucose catabolism provides a rapid means to generate ATP and boosts the pentose phosphate pathway (PPP) and tricarboxylic acid (TCA) cycle for the generation of important immunometabolites such as NADPH, itaconate, and prostaglandins (1–3). On the other hand, cytokines such as interleukin 4 (IL-4) induce an anti-inflammatory phenotype in macrophages important for tissue homeostasis and antiparasitic responses. These alternatively activated macrophages have increased fatty acid β-oxidation (FAO) and OXPHOS (1). The majority of studies on macrophage metabolism focus on IFN-γ/LPS- and IL-4-induced states, although accumulating evidence suggests that macrophage polarization states are multidimensional (4). The metabolic characterization of more-diverse macrophage phenotypes and their impact on antimicrobial capacity in the context of specific infections are largely unexplored.

Recent studies also highlight a link between Mycobacterium tuberculosis (Mtb) pathogenesis and host metabolism. Mtb is the causative agent of tuberculosis (TB), which kills more people yearly than any other infection. Macrophages are a main cellular niche of Mtb (5, 6). Within the lung, alveolar macrophages (AMs) and interstitial macrophages (IMs) are the major populations of infected macrophages, and they have distinct metabolic profiles. AMs, which preferentially utilize FAO, represent a permissive niche for Mtb replication, whereas glycolytically active IMs restrict infection (7). Mtb alters macrophage metabolism along with shifting macrophage phenotype to a more proinflammatory state (8–12). Mtb enhances the dependency of mitochondrial oxidative metabolism on fatty acids, in particular exogenous fatty acids, and induces the formation of lipid-droplet-filled or “foamy” macrophages (11, 13–15). Foamy macrophages are found within the inner layers of granulomas, a common histopathologic feature of TB, and the bacilli themselves can be found in close approximation to intracellular lipid droplets. It is thought that lipid bodies serve as a source of nutrients in the form of cholesterol esters and fatty acids for the bacilli, thus providing a hospitable niche for the bacterium (14–20). Lipid accumulation by Mtb in this environment may also promote a nonreplicating phenotype (16). Moreover, a growing body of literature suggests a link between lipid metabolism and cellular control of Mtb. A number of host-directed therapies (HDTs) under investigation for TB, such as statins and metformin, modulate host lipid metabolism (21, 22). However, how these metabolic shifts influence the outcome of Mtb infection remains incompletely understood.

Cellular regulators of lipid metabolism such as microRNA-33 (miR-33) and the transcription factors peroxisome proliferator-activated receptor α (PPARα) and PPAR-γ play a role in the formation of Mtb-induced lipid droplets. Studies in which miR-33, PPAR-α, and PPAR-γ were modulated revealed a correlation between cellular lipids and intracellular survival of mycobacteria, such that increased intracellular lipids were associated with enhanced bacterial replication (12, 23, 24). These studies led to the idea that impaired host fatty acid catabolism and the formation of foamy macrophages serves to enhance intracellular survival of Mtb (12, 23, 24), but direct evidence is lacking, and other studies support the idea that host lipid droplets promote host defense (25). Because miR-33, PPAR-α, and PPAR-γ impact diverse aspects of host biology that influence the antimicrobial capacity of macrophages, including mitochondrial function and autophagy, a causal link between the lipid changes they induce and the survival benefit to the bacilli has not been clearly established. Previously, we showed that induction of miR-33/33* in response to Mtb enhances intracellular survival of Mtb (12). We showed that this probacterial function of miR-33/33* was related in part to its ability to block autophagy, and we speculated that its ability to block fatty acid catabolism and promote the formation of lipid droplets also enhanced bacterial replication. Here, we tested whether blocking fatty acid catabolism indeed promotes Mtb replication. Instead, we found that inhibiting macrophage FAO chemically or genetically restricted intracellular growth of Mtb. FAO inhibition promoted a pathway of bacterial killing in which induction of mitochondrial reactive oxygen species (mROS) led to NADPH oxidase and autophagy-dependent control of Mtb.

## RESULTS

### Inhibiting FAO restricts growth of intracellular Mtb.

**FIG 1:**
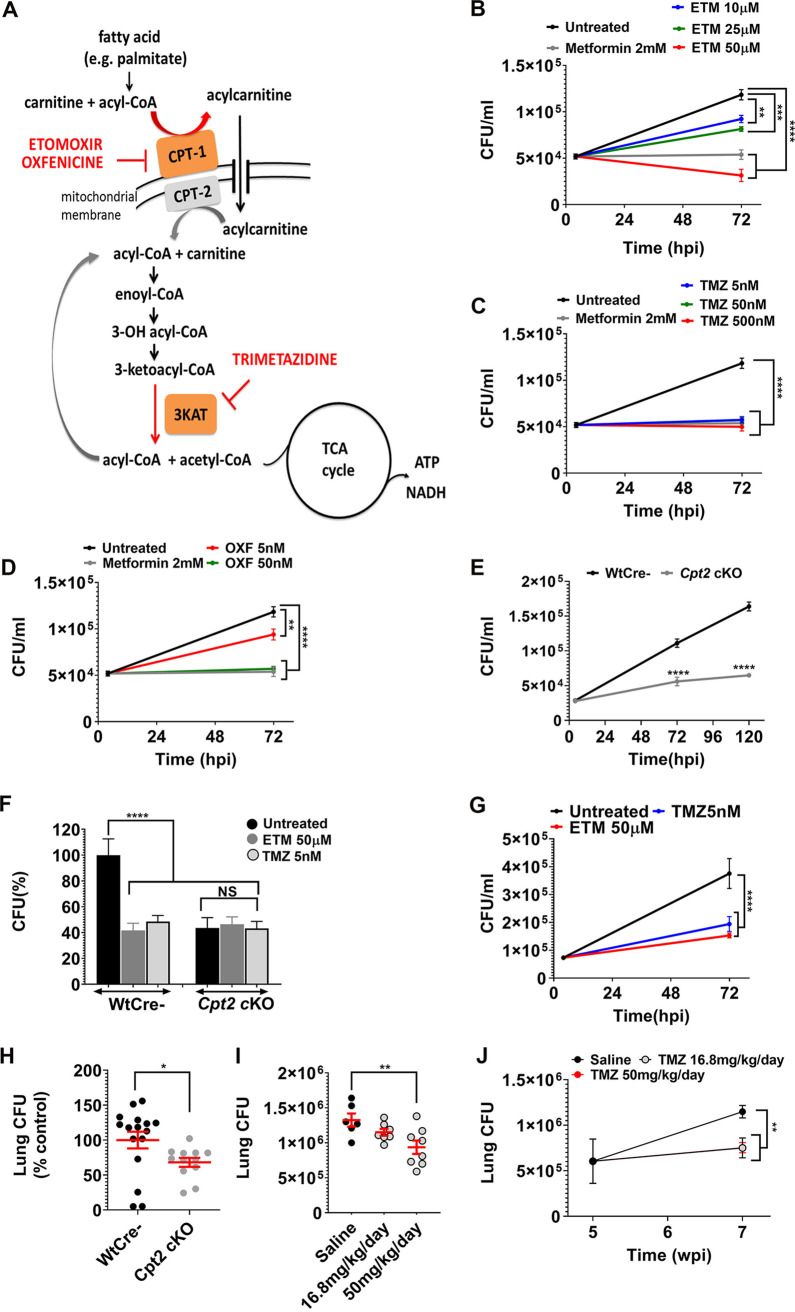
Inhibition of macrophage FAO restricts intracellular Mtb growth. (A) In the carnitine shuttle, long-chain fatty acids that have been activated to acyl-CoA derivatives are converted to acylcarnitines by CPT1 at the outer mitochondrial membrane. Etomoxir and oxfenicine inhibit CPT1. Acylcarnitines are transported across the mitochondrial membrane by a dedicated translocase. In the mitochondria, CPT2 converts acylcarnitines back to acyl-CoA and carnitine. Acyl-CoA chains then undergo β-oxidation, successively generating acetyl-CoA that enters the TCA cycle. Trimetazidine inhibits 3-ketoacyl-CoA thiolase, which catalyzes the release of acetyl-CoA from the acyl-CoA chain. (B to D) Survival of Mtb (H37Rv) in BMDMs that were untreated or treated for 72 h with 2 mM metformin (MET) or indicated concentrations of etomoxir (ETM), (B), trimetazidine (TMZ) (C), or oxfenicine (OXF) (D). (E) Survival of Mtb in BMDMs from *Cpt2* KO (*Cpt2^fl/fl^* LysM-Cre^+^) and littermate controls (*Cpt2^fl/f^* Cre-, wild type [Wt]) 4, 72, and 120 hpi. (F) Survival of Mtb in BMDMs from *Cpt2* cKO and Wt controls treated with ETM, TMZ, or vehicle control for 72 h. CFU are expressed as a percentage of the WT untreated control. (G) PMA-differentiated THP-1 cells were infected with Mtb and treated with ETM or TMZ for 72 hpi prior to enumerating CFU. In panels B to G, data show means ± standard error of means (SEM) (error bars) from at least two independent experiments. Statistical significance by one-way ANOVA (B to D, F, and G) or unpaired Student’s *t* test with Welch’s correction (E) is indicated as follows: **, *P* ≤ 0.009; ***, *P* = 0.0003; ****, *P* < 0.0001; NS, not significant. (H) *Cpt2* cKO mice and littermate controls were infected by aerosol with 100 to 200 Mtb CFU per animal, and lung CFU were enumerated 7 days postinfection. Data are expressed as a percentage of the control value from two independent experiments. Data are means ± SEM. *, *P* = 0.01 calculated using Mann-Whitney test. (I) TMZ was tested in mice that were infected with Mtb (day 1 CFU = 3,552) and treated for the first 2 weeks of infection at the indicated doses. Plots show mean lung CFU after 2 weeks of TMZ treatment (16.8 or 50 mg/kg/day). **, *P* = 0.02 calculated with Mann-Whitney test. (J) Mice were infected by aerosol with 155 Mtb for 5 weeks and then were treated with TMZ or saline control for 2 weeks, and total lung CFU were enumerated. **, *P* ≤ 0.005 calculated using Mann-Whitney test. See also Fig. S1 and S2 in the supplemental material. wpi, week postinfection.

miR-33 inhibits FAO by targeting genes such as carnitine palmitoyltransferase 1 (*CPT1*) and the hydroxyacyl-coenzyme A (CoA) dehydrogenase trifunctional multienzyme complex subunit beta (HADHB). CPT1 is required for the entry of long-chain fatty acids into the mitochondrial matrix, while HADHB catalyzes the final step of β-oxidation. Small-molecule inhibitors, etomoxir (ETM), oxfenicine (OXF), and trimetazidine (TMZ) also target these steps in FAO. ETM and OXF inhibit CPT1, whereas TMZ blocks the 3-ketoacyl-CoA thiolase activity of HADHB (Fig. 1A) (26). Thus, to determine whether the inhibition of FAO conferred by miR-33 contributed to its ability to enhance the intracellular survival of Mtb, we tested the effect of chemical inhibition of FAO on intracellular bacterial replication. We infected murine bone marrow-derived macrophages (BMDMs) with the H37Rv strain of Mtb, and 4 h later, we washed the cells to remove extracellular bacilli and supplemented the media with ETM, OXF, or TMZ. After treatment, we estimated intracellular Mtb growth by plating for CFU at 72 h postinfection (hpi). Unexpectedly, treatment with all three FAO inhibitors restricted the intracellular growth of Mtb compared to untreated controls. We observed a dose-dependent reduction in Mtb CFU in macrophages treated with micromolar concentrations of ETM (Fig. 1B), while TMZ and OXF were effective at nanomolar concentrations (Fig. 1C and D; see also Fig. S1A in the supplemental material). By comparison, metformin (MET), which has previously been shown to restrict intracellular Mtb, worked at millimolar concentrations (22) (Fig. 1B to D). The antitubercular activity of TMZ was corroborated using a “live-dead” reporter strain of Mtb (Fig. S1B). This strain constitutively expresses mCherry and expresses green fluorescent protein (GFP) under the control of a tetracycline-inducible promoter (27). TMZ reduced the number of GFP-positive bacteria, consistent with fewer metabolically active or live bacilli compared to controls. Using calcein fluorescent dye, we found that macrophage viability was unaffected by FAO inhibitors ETM and TMZ (Fig. S1C). In addition, the inhibitors did not have direct toxicity on Mtb in broth culture (Fig. S1D and S1E), and they enhanced macrophage control against a distantly related mycobacterium, Mycobacterium abscessus (Fig. S1F). These assays will underestimate the effect of host-directed therapies on intracellular bacteria, since HDTs do not directly kill extracellular bacteria, which are not completely removed by washing. Consistent with the ability to impair FAO, TMZ enhanced cellular lipid accumulation based upon BODIPY (4,4-difluoro-1,3,5,7,8-pentamethyl-4-bora-3a,4a-diaza-s-indacene) staining (Fig. S1G). In addition, in keeping with impaired FAO, TMZ-treated macrophages had reduced oxygen consumption (Fig. S1H), similar to macrophages from mice that were genetically deficient in FAO due to deletion of *Cpt2* (*Cpt2^fl/fl^* LysM-Cre^+^*; Cpt2* cKO [*Cpt2* knocked out]) (28).

10.1128/mBio.01139-20.1 FIG S1 Inhibition of macrophage FAO restricts intracellular M. tuberculosis growth. (A) Mtb survival in murine BMDMs that were either untreated or treated with 0.1 nM and 0.5 nM TMZ for 4 days. Data show means ± SD from six replicates with a *P* value of 0.007 (**) and not significant (NS) calculated using one-way ANOVA. (B) Murine BMDMs were infected with live-dead reporter expressing H37Rv and were either untreated or treated with 50 nM TMZ for 6 days. The reporter Mtb strain constitutively expresses mCherry and has GFP controlled by a tetracycline-inducible promoter. To induce GFP expression, 200 nM anhydrotetracycline was added 2 days after infection. Metabolically active bacteria express both GFP and mCherry, whereas dead bacteria are positive for only mCherry. Representative images show more dead Mtb upon TMZ treatment compared to the control. Scale bar = 10 μm. (C) Macrophages were uninfected or infected with Mtb for 72 h, and macrophage cell viability was assessed using calcein dye mean fluorescence intensity (MFI). The plot shows cell viability expressed as a percentage of the value for uninfected cells. Data show means ± SEM. (D) H37Rv expressing the Vibrio harveyi luciferase (Rv-lux) was cultured in 7H9 broth with or without 1 mM TMZ for 7 days. The plot shows bacterial growth as assessed by relative luminescence units (RLU). (E) Minimal inhibitory concentrations (MICs) of etomoxir, oxfenicine, metformin, and isoniazid (INH) (positive control) was determined by culturing H37Rv for 4 days in 7H9 broth supplemented with various doses of inhibitors and measuring absorbance at 600 nm. (F) BMDMs were infected with M. abscessus that were untreated or treated with indicated concentrations of amikacin or TMZ for 48 h. Intracellular growth of bacilli was measured by CFU. Data show means ± SEM. ****, *P* ≤ 0.0001 by ordinary one-way ANOVA. (G) BODIPY fluorescent dye was used to stain lipid bodies in BMDMs that were infected for 24 h with Mtb and either untreated or treated with TMZ. Shown here are means ± SEM of the percent area in a cell occupied by BODIPY stain. ****, *P* ≤ 0.0001 by unpaired Student’s *t* test with Welch’s correction. (H and I) Using extracellular flux analysis, we quantified the oxygen consumption rate (OCR) (H) and extracellular acidification rate (ECAR) (I) in uninfected control and *Cpt2* cKO BMDMs treated with 5nM TMZ or solvent control for 3 h, followed by sequential addition of oligomycin (A), FCCP (B), and rotenone plus antimycin (C). Data show averages ± SD for 16 replicates. Download FIG S1, PDF file, 0.1 MB. Copyright © 2020 Chandra et al. 2020 Chandra et al. This content is distributed under the terms of the Creative Commons Attribution 4.0 International license.

10.1128/mBio.01139-20.2 FIG S2 Pharmacokinetics study of TMZ in mice. The half-life of TMZ was determined in C57BL/6 mice that were administered the compound (A) orally at 15 mg/kg or (B) intravenously at 3 mg/kg. (C) TMZ (10.66 mg/kg/day) was administered to female C57BL/6 mice (*n* = 5) for 48 h using Alzet osmotic pumps and achieved an average concentration (*C*_ave_) of 34.5 ng/ml. (D) Protein binding of TMZ in mouse plasma (C57BL/6) was measured using rapid equilibrium dialysis (*n* = 3). Warfarin was used as a control. In humans, TMZ is reportedly weakly protein bound (∼16%) (31). Serum concentrations of TMZ were estimated for the two-week, acute infection model after 14 days of treatment (E) and the infection model in which mice were treated after 5 weeks of infection (F); drug levels were measured on day 5 (▲) and 14 (○) of treatment. Values are means ± SEM. Download FIG S2, PDF file, 0.1 MB. Copyright © 2020 Chandra et al. 2020 Chandra et al. This content is distributed under the terms of the Creative Commons Attribution 4.0 International license.

To confirm that the antimycobacterial activity was indeed a result of FAO inhibition, we compared the intracellular growth of Mtb in *Cpt2* cKO macrophages to littermate controls. As shown in Fig. 1E, Mtb was significantly impaired in *Cpt2* cKO macrophages compared to control. Moreover, FAO inhibitors lacked activity in *Cpt2* cKO macrophages, confirming their target specificity (Fig. 1F). To determine whether FAO inhibitors were also active against Mtb in human macrophages, we treated phorbol 12-myristate 13-acetate (PMA)-differentiated THP-1 cells. As we found in murine macrophages, FAO inhibitors impaired Mtb growth in THP-1 cells (Fig. 1G). Taken together, our findings suggest that FAO inhibition enhances the ability of macrophages to control Mtb infection. This enhanced host-mediated control could reflect a shift to a metabolically inactive, nonreplicating bacterial state or enhanced bacterial killing. Since ETM is documented to have off-target effects (29) and TMZ was effective at nanomolar concentrations, we selected TMZ and *Cpt2* cKO mice for further study.

Given our *in vitro* findings, we investigated whether TMZ impacts mycobacterial control in mice. We compared Mtb infection in mice with myeloid-specific knockout of *Cpt2* (*Cpt2* cKO) and littermate controls. We exposed mice to Mtb by aerosol and estimated lung bacterial burden after 7 days, when the pathogen exclusively infects alveolar macrophages, which are permissive to Mtb growth and rely more on fatty acid oxidation than glycolysis (6, 7). We observed that growth of Mtb was significantly reduced in *Cpt2* cKO mice compared to controls (Fig. 1H). To determine whether chemical inhibition of FAO had antimicrobial activity in mice, we used Alzet osmotic pumps to deliver a continuous dose over 2 weeks (Fig. S2A to D). In an acute infection model, we treated mice with TMZ during the first 2 weeks of infection, which represents the early innate immune phase in which there is rapid Mtb replication (30). We also initiated TMZ treatment after 5 weeks of infection, after the onset of adaptive immunity. In both cases, TMZ treatment modestly reduced bacterial burdens in the lungs (Fig. 1I and J). TMZ is approved by the European Medicines Agency to treat angina, and pharmacokinetic (PK) analysis demonstrated that serum TMZ concentrations in the infected mice were similar to those achieved clinically (Fig. S2E and F). In addition, consistent with the extremely wide therapeutic index of TMZ in preclinical toxicology studies (31), no treatment-related morbidity or mortality was observed. Overall, we conclude that inhibiting host fatty acid metabolism modestly reduces mycobacterial burden *in vivo*.

### FAO inhibition triggers mitochondrial ROS.

**FIG 2:**
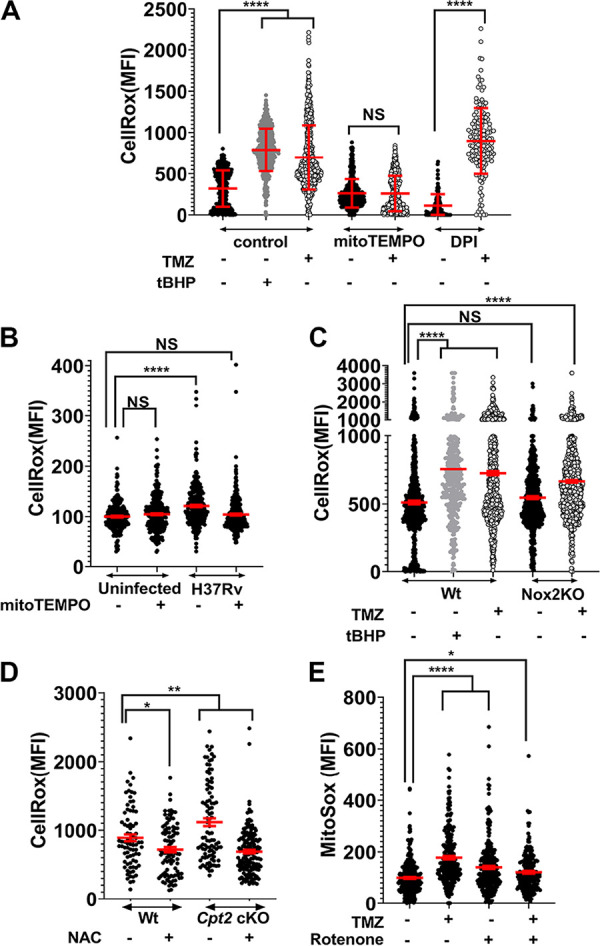
FAO inhibition induces mitochondrial ROS. The CellRox mean fluorescence intensity (MFI) of BMDMs with various treatments is shown. (A) Uninfected iBMDMs that were untreated (-) or treated (+) with 500 nM TMZ for 3 h, alone or in the presence of mitoTEMPO (10 μM) or DPI (10 mM). (B) Uninfected and infected BMDMs with or without mitoTEMPO (10 μM) 4 hpi. (C) Mtb-infected Wt and *Nox2* KO BMDMs that were untreated or treated with 5 nM TMZ for 3 h. (A and C) *tert*-Butyl hydroperoxide (tBHP; 0.25 mM, 30 min) was used as a positive control. (D) Mtb-infected *Cpt2* cKO and control BMDMs 24 hpi that were untreated or treated with *N*-acetyl cysteine (NAC) (10 mM). (E) MitoSox MFI of uninfected BMDMs that were untreated or treated with 500 nM TMZ for 3 h, alone or in the presence of rotenone (10 μM, 30 min). Data show means ± standard deviations (SD) of duplicates (A) and means ± SEM (B to E) from at least two independent experiments where MFI was derived from more than 100 individual cells. *, *P* = 0.02; **, *P* = 0.001; ****, *P* ≤ 0.0001; NS, not significant, calculated using unpaired Student’s *t* test with Welch’s correction (A) and ordinary one-way ANOVA (B to E). For panels B and E, values are expressed as percentage of the value for the uninfected, untreated cells. See also Fig. S3 and S4.

We turned to *in vitro* studies to assess how host fatty acid metabolism was influencing control of Mtb. Since TMZ targets an enzyme in the mitochondria (32), a major site of reactive oxygen species (ROS) production, we examined whether FAO inhibition promotes ROS production. We treated uninfected immortalized BMDMs (iBMDMs) with TMZ and observed an increase in ROS as early as 3 h after treatment (Fig. 2A). Addition of mitoTEMPO, a mitochondrial ROS (mROS) scavenger, abolished this ROS burst, whereas diphenyleneiodonium chloride (DPI), which inhibits NADPH oxidase, had no effect, suggesting that the ROS was induced in the mitochondria (Fig. 2A). Toll-like receptor activation and infection with Gram-negative bacteria have previously been shown to result in mROS production (33, 34), and Mtb infection induced a small amount of mROS (Fig. 2B). However, significantly more ROS was generated in Mtb-infected BMDMs after TMZ treatment and was dose dependent (Fig. 2C and Fig. S3A). The TMZ-induced ROS still occurred in macrophages that lacked the NADPH oxidase (*Nox2* KO), consistent with a mitochondrial source (Fig. 2C). ROS levels in Mtb-infected *Cpt2* cKO BMDMs were also significantly higher than in control macrophages (Fig. 2D). We confirmed that TMZ induced mROS by using MitoSox fluorescent dye (Fig. 2E). Combined, these results demonstrate that TMZ induces ROS from a mitochondrial source after 3 h of treatment in both infected and uninfected macrophages.

10.1128/mBio.01139-20.3 FIG S3 FAO inhibition promotes oxidative stress and xenophagy activation. (A) Comparison of mROS levels in uninfected BMDMs that were untreated or treated with 0.1 nM TMZ or 0.5 nM TMZ for 3 h. Antimycin was used as positive control. Values are means ± 95% confidence interval (95% CI). *, *P* = 0.04; **, *P* = 0.007 calculated using one-way ANOVA from 100 to 150 cells. (B) Mtb survival in BMDMs that were untreated or treated with 5 nM TMZ, with or without mitoTEMPO, 72 hpi. Values are means ± SEM from two independent experiments with 17 to 20 replicates. The *P* values were calculated using one-way ANOVA. (C) Intracellular survival of H37Rv and Δ*katG* mutant in BMDMs that were untreated or treated with FAO inhibitors, 4 and 120 hpi. Values are means ± SEM from two independent experiments with at least 25 replicates. *P* values were calculated using one-way ANOVA. (D) IF images and plot showing MFI of LC3 (green) colocalized with intracellular Mtb (red) in BMDMs that were untreated or treated with 5 nM TMZ, with or without mitoTEMPO, for 24 h. Values are means ± 95% CI from 100 to 200 bacilli. ****, *P* < 0.0001 calculated using one-way ANOVA. Here, rapamycin (50 nM, 24 h) was used as a positive control. (E) MFI of p62 colocalized with Mtb was compared in BMDMs that were untreated or treated with 0.1 nM or 5 nM TMZ for 24 h. Values are means ± 95% CI from 100 to 200 bacilli. *P* values were calculated using Student’s *t* test with Welch’s correction. Download FIG S3, PDF file, 0.2 MB. Copyright © 2020 Chandra et al. 2020 Chandra et al. This content is distributed under the terms of the Creative Commons Attribution 4.0 International license.

10.1128/mBio.01139-20.4 FIG S4 Effect of TMZ on proinflammatory cytokine secretion by macrophages. Supernatants from uninfected or Mtb-infected BMDMs that were untreated or treated with indicated concentrations of TMZ were harvested 24 and 72 hpi. The levels of proinflammatory cytokines and chemokines TNF-α (A), IL-6 (B), CXCL2 (C), IFN-β (D), CCL2 (E), and CXCL10 (F) were measured using a Milliplex MAP Mouse Cytokine/Chemokine Magnetic Bead Panel. *, *P* ≤ 0.02; **, *P* = 0.004 calculated using ordinary one-way ANOVA. Download FIG S4, PDF file, 0.1 MB. Copyright © 2020 Chandra et al. 2020 Chandra et al. This content is distributed under the terms of the Creative Commons Attribution 4.0 International license.

We hypothesized that FAO inhibition resulted in mROS generation because of perturbed electron flow within the electron transport chain (ETC). ROS can be generated in multiple sites along the ETC during forward electron transport, as well as when electrons flow in reverse through complex I (NADH:coenzyme Q reductase) (35). To determine the site within the ETC where TMZ induced ROS, we measured mROS production in macrophages treated with TMZ in combination with rotenone, an inhibitor of complex I. When electrons are flowing in the forward direction, rotenone prevents electron transport to coenzyme Q (CoQ), resulting in ROS generation. In contrast, during reverse electron transport (RET), rotenone reduces ROS by preventing CoQ from transferring electrons back to complex I, where the RET-ROS is generated. We treated uninfected BMDMs with TMZ for 3 h, and 30 min prior to measuring mROS, we added rotenone. As expected, rotenone and TMZ on their own increased mROS production. In contrast, rotenone decreased the amount of ROS generated in response to TMZ treatment (Fig. 2E). These findings are consistent with the idea that under conditions of TMZ treatment there is enhanced ROS generated due to RET at complex I. Overall, we conclude that FAO inhibition promotes mROS production from the ETC.

Since recent studies have shown that mROS contributes to microbial control in macrophages (33, 36), we asked whether TMZ-induced mROS was important for infection control or simply a by-product of metabolic perturbations. To address this, we estimated Mtb burden in macrophages treated with TMZ alone or in combination with mitoTEMPO. As shown in Fig. S3B, mitoTEMPO on its own had little effect on the infection, but it partially reversed the antimicrobial effect of TMZ treatment. This suggested that mROS contributed to the host control established by TMZ treatment, although there may be other contributing factors or mitoTEMPO may not neutralize mROS for the prolonged conditions of the assay. We also considered that TMZ treatment altered cytokine-driven antimicrobial responses, but we did not observe differences in the production of tumor necrosis factor alpha (TNF-α), IL-6, CXC chemokine ligand 2 ( CXCL2), IFN-β, CC chemokine ligand 2 (CCL2), and CXCL10 in response to infection and TMZ treatment (Fig. S4A to F). In addition, we considered that inhibition of FAO might lead to a corresponding increase in glycolysis, which has been linked to antimicrobial responses, but Seahorse analysis suggested reduced, not increased, extracellular acidification rates (ECARs) after TMZ treatment (Fig. S1I). Overall, we conclude that FAO inhibition promotes mROS production from the ETC, which contributes to macrophage control of Mtb infection.

### FAO inhibition-induced mitochondrial ROS drives NADPH oxidase recruitment to phagosomes.

**FIG 3:**
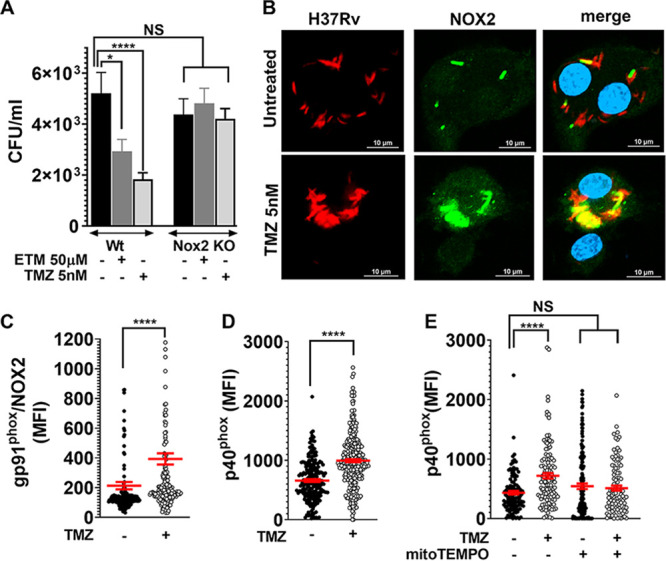
FAO inhibition promotes NADPH oxidase recruitment on Mtb phagosomes. (A) Survival of Mtb in BMDMs from *Nox2* KO and control mice that were untreated or treated with the indicated concentrations of ETM and TMZ, 120 hpi.*, *P* = 0.04; ****, *P* = 0.0001, ordinary one-way ANOVA. (B) Immunofluorescence (IF) microscopy of gp91^phox^/NOX2 (green) and dsRed-expressing H37Rv (red) in BMDMs that were infected and treated with 5 nM TMZ or untreated for 24 h. The colocalized region is shown in yellow. Bars = 10 μm. (C and D) MFI of the NADPH oxidase subunits gp91^phox^/NOX2 (C) and p40^phox^ (D) colocalized with H37Rv in BMDMs that were untreated or treated with 5 nM TMZ (24 hpi). (E) MFI of p40^phox^ colocalized with Mtb in BMDMs treated with 5 nM TMZ alone or in the presence of mitoTEMPO (48 hpi). (C to E) Automated image analysis was used to quantify gp91^phox^/NOX2 or p40^phox^ MFI colocalized with more than 100 bacilli in at least two independent experiments. Data show means ± SEM of MFI from around individual bacilli in the sample. *, *P* = 0.02, ****, *P* ≤ 0.0001 calculated using unpaired Student’'s *t* test with Welch’s correction (C and D) or ordinary one-way ANOVA (E).

Previous studies suggest a link between mROS and NADPH oxidase that contributes to macrophage defense (37). Indeed, while 3 h of TMZ treatment significantly increased mROS in both wild-type (WT) and *Nox2* KO BMDMs (Fig. 2C), we observed that the anti-Mtb activity of FAO inhibitors required NADPH oxidase (Fig. 3A). This suggested that the antimycobacterial activity in FAO-inhibited macrophages actually depended upon both a mitochondrial source and NADPH oxidase. Normally, pathogen-associated molecular patterns (PAMPs) promote the recruitment of the NADPH oxidase to microbial phagosomes immediately after phagocytosis (38). However, Mtb impairs the recruitment of the NADPH oxidase to the mycobacterial phagosome (39, 40). Remarkably, at 24 hpi, we observed that TMZ increased the recruitment of NADPH oxidase subunits gp91^phox^/NOX2 and p40^phox^ to the mycobacterial phagosomes (Fig. 3B to D). Moreover, we found that NADPH oxidase recruitment was dependent on TMZ-induced mROS, as it was reversed with the addition of mitoTEMPO (Fig. 3E). Thus, FAO inhibition appears to enhance two antimicrobial responses that are suboptimal during Mtb infection, mROS production and phagosomal recruitment of the NADPH oxidase. Mitochondria alone contributed to ROS within 3 h of FAO inhibition, while the role of NADPH oxidase was appreciable at later time points, and enhanced macrophage control of Mtb depended upon both mROS and the NADPH oxidase.

### FAO inhibition promotes xenophagy to restrict intracellular Mtb growth.

**FIG 4:**
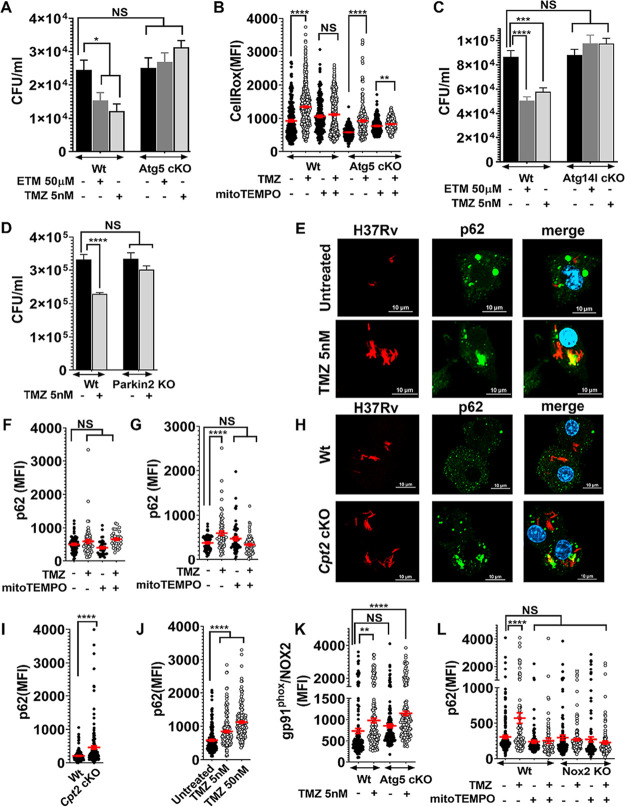
Antimicrobial activity of FAO inhibitors depends on autophagy. (A) Survival of Mtb was compared in FAO inhibitor-treated control and *Atg5* cKO macrophages, 72 hpi. Data show means ± SEM with *, *P* ≤ 0.03 calculated using one-way ANOVA. (B) CellRox MFI in Mtb-infected control and *Atg5* cKO BMDMs after TMZ treatment (5 nM, 3 h), with or without mitoTEMPO. **, *P* = 0.002; ****, *P* ≤ 0.0001 by unpaired Student's *t* test with Welch’s correction. (C and D) Survival of Mtb in BMDMs from control and *Atg14l* cKO cells 72 hpi (C) and *Parkin2* KO cells 120 hpi. (D) Data show means ± SEM. ***, *P* = 0.0002; ****, *P* ≤ 0.0001 using ordinary one-way ANOVA in panels C and D. (E) IF microscopy of p62 (green) and dsRed-expressing H37Rv (red) in BMDMs treated with 5 nM TMZ 24 hpi. The colocalized region is shown in yellow. Bars = 10 μm. (F and G) MFI of p62 colocalized with Mtb was measured in BMDMs treated with 5 nM TMZ alone or with mitoTEMPO for 3 h (F) or 24 h (G). ****, *P* ≤ 0.0001 by ordinary one-way ANOVA. (H and I) IF images (H) and MFI (I) of p62 colocalized with Mtb in *Cpt2* KO and control BMDMs 24 hpi. (J) MFI of p62 colocalized with Δ*esxA* mutant in BMDMs treated with TMZ for 24h. (K) MFI of gp91^phox^/NOX2 colocalized with Mtb in control versus *Atg5* cKO BMDMs after TMZ treatment (5 nM, 24 hpi). (L) MFI of p62 colocalized with Mtb in control and Nox2KO macrophages treated with 5 nM TMZ with or without mitoTEMPO, 24 hpi. Data show means ± SEM.**, *P* = 0.001; ****, *P* ≤ 0.0001 calculated using unpaired Student’s *t* test with Welch’s correction in panel K and ordinary one-way ANOVA for panels J and L. All panels show data from one representative experiment from at least two independent replicates. See also Fig. S3.

ROS might be directly cytotoxic to bacteria or might promote a nonreplicating state. For example, macrophage-derived ROS induces a metabolically inactive, drug-tolerant state in Staphylococcus aureus (41). To determine whether ROS was likely to have direct antibacterial effect, we examined the impact of FAO inhibitor treatment on an Mtb mutant that lacks the catalase-peroxidase KatG. If TMZ-induced ROS was directly antibacterial, we anticipated that *katG* mutants would be more susceptible. However, we found no difference in the antimicrobial activity of TMZ between WT and *katG* mutants (Fig. S3C). ROS generated by the NADPH oxidase has also been shown to promote a trafficking pathway called LC3-associated phagocytosis (LAP). Both LAP and autophagy of microbes (xenophagy) are characterized by the association of lipidated LC3 (LC3-II) with microbe-containing vacuoles (42). LAP and xenophagy depend upon certain common ATG (autophagy-related) proteins, and they also have unique requirements. To determine whether either LC3-trafficking pathway was involved in the antimicrobial activity of TMZ, we compared the efficacy of TMZ between control and *Atg5* cKO macrophages, which are deficient in both LAP and xenophagy. Indeed, the antimycobacterial control established by FAO inhibition was reversed in *Atg5*-deficient macrophages (Fig. 4A). Although the baseline ROS staining was lower in *Atg5* cKO macrophages compared to control, ROS was still induced by TMZ in *Atg5* cKO macrophages, suggesting that ATG5 acts downstream of mitoROS production (Fig. 4B). To distinguish xenophagy from LAP, we examined *Atg14l* cKO and *Parkin2* KO macrophages, which are specifically involved in xenophagy. As we had seen in *Atg5* cKO macrophages, the antimycobacterial control established by FAO inhibition was reversed in *Atg14l* cKO and *Parkin2* KO as well (Fig. 4C and D). In addition, in WT macrophages, TMZ treatment resulted in enhanced colocalization between Mtb and the autophagy adaptor, p62, and LC3 (Fig. 4E and Fig. S3D). The enhanced colocalization between Mtb and p62 occurred after 24-h treatment, was dependent on mROS, and was dose dependent (Fig. 4F and G and Fig. S3E). Combined, these data demonstrate that inhibition of FAO promotes xenophagy to control intracellular Mtb growth. Indeed, autophagosomal targeting of Mtb was also inherently higher in *Cpt2* cKO macrophages than in WT controls (Fig. 4H and I). Moreover, TMZ treatment facilitated xenophagy of a mutant Mtb strain lacking *esxA* (Δ*esxA*), which is unable to perforate phagosomes and usually does not activate xenophagy (Fig. 4J).

**FIG 5:**
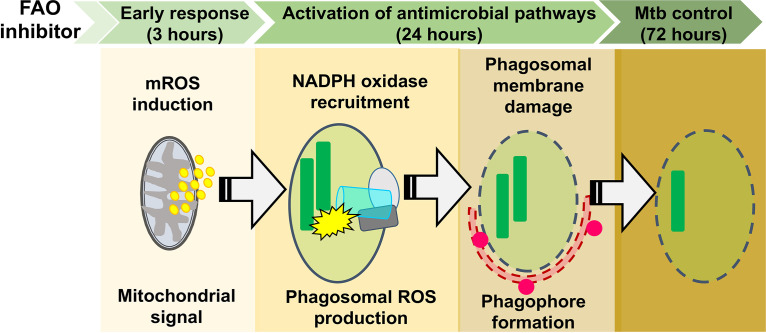
Schematic of the antimycobacterial mechanism of FAO inhibitors. FAO inhibition induces mROS burst, which promotes the recruitment of the NADPH oxidase to the phagosomal membrane. Phagosomal ROS promotes subsequent p62 and LC3 colocalization, perhaps as a result of phagosomal membrane damage, leading to enhanced control of Mtb.

These studies established that FAO inhibition induced mROS, which promoted NADPH oxidase recruitment to the Mtb phagosome and xenophagy. Next we examined the relationship between the NADPH oxidase and xenophagy. We observed that the enhanced NADPH oxidase recruitment in response to TMZ occurred independently of autophagy, as it was also seen in *Atg5* cKO macrophages (Fig. 4K). On the other hand, TMZ failed to promote xenophagy in NADPH oxidase-deficient macrophages (Fig. 4L). Taken together, our data show that mROS was the primary signal induced by FAO inhibition that resulted in enhanced NADPH oxidase and xenophagy, which promoted bacterial control. Figure 5 shows a model for the sequential events resulting in the antimycobacterial activity of FAO inhibitors.

## DISCUSSION

Recent work has highlighted the relationship between macrophage metabolism and inflammatory phenotype (43). Mtb-induced glycolysis in macrophages reduces intracellular bacterial survival through enhanced IL-1β production, but the impact of fatty acid metabolism is not well established. A prevailing idea is that increased cellular lipids provide a nutrient source for bacterial replication (12, 23, 24), although lipid droplets may also promote a nonreplicating phenotype of the bacilli and host immunity (16, 25). We found that inhibition of host FAO, which enhanced lipid body formation in macrophages, impaired mycobacterial replication, which could reflect promoting a nonreplicating phenotype or enhanced bacterial killing. We established the antimicrobial mechanism of FAO inhibition using specific inhibitors and genetically FAO-deficient *Cpt2* cKO macrophages. Unexpectedly, we found that blocking FAO enhanced macrophage control of Mtb by eliciting mROS, which promoted NADPH oxidase and xenophagy to restrict Mtb replication. Thus, our results identify mROS as a link between macrophage fatty acid metabolism and macrophage effector functions.

How might inhibition of FAO generate mROS? Typically, FAO produces NADH and FADH_2_, which are oxidized by respiratory chain supercomplexes to generate forward electron flow required for ATP production. The respiratory chain is organized into interacting supercomplexes to minimize leakage of electrons and ROS formation. A recent study reports that enzymes of FAO physically interact with the NADH-binding domain of complex I of the electron transport chain (ETC), forming an integrated, multifunctional complex (44). Thus, in the absence of NADH shuttling from FAO through these supercomplexes, reverse electron transport (RET) might occur. Combined treatment with TMZ and rotenone, a complex I inhibitor, decreased mROS levels, which is consistent with the idea that RET at complex I is the source of mROS generated by TMZ treatment. Toll-like receptor signaling also alters supercomplex assembly and generates RET-ROS at complex I, both of which impact immunological signaling (33, 34, 37, 45). Thus, the mROS induced by TMZ may activate an antimicrobial pathway that is normally induced by infection and protective, but suboptimal during Mtb infection. mROS has also been shown to contribute to macrophage control of Streptococcus pneumoniae, Staphylococcus aureus, and Salmonella enterica serotype Typhimurium (33, 36, 46, 47). Our results suggest that mROS does not exert its antimicrobial function by directly impairing Mtb growth, since mROS was generated in macrophages lacking *Nox2* and *Atg5*, but the antimycobacterial activity was lost. In addition, Δ*katG* mutants were not hypersusceptible to TMZ treatment. Rather, ROS appears to serve as a signal that enhances LC3 trafficking. A stimulatory effect of mROS on the NADPH oxidase has been documented in nonlymphoid cells, and recent work shows that in neutrophils, mROS activates the NADPH oxidase and other effector functions (48–50). How mROS regulates phagosomal NADPH oxidase recruitment and assembly will be important to establish, as our work indicates, unexpectedly, that phagosomal NADPH oxidase can be augmented, even late after bacterial uptake, to promote microbial clearance of intracellular bacilli. This might be a way to clear foci of persistent bacilli.

In addition to NADPH oxidase recruitment, mROS also promoted xenophagy. Xenophagy and LAP are related LC3 trafficking pathways that can promote microbial clearance (51). They also differ, as LAP involves LC3 recruitment to a single phagosomal membrane, whereas xenophagy depends upon formation of a double-membrane compartment. We found that the antimicrobial activity of TMZ depended on ATG14L and PARKIN, which are required for canonical autophagy and Mtb xenophagy (52). In addition, TMZ enhanced the association of phagosomes with p62, a selective autophagy adaptor, and the response occurred well after bacterial uptake, all of which suggest that the mechanism of clearance is xenophagy and not LAP, despite the involvement of NADPH oxidase which has been linked to LAP. One possibility is that both LAP and xenophagy are enhanced by FAO inhibition. Alternatively, the NADPH oxidase might generate phagosomal membrane damage, which is known to promote pathogen ubiquitination, adaptor recruitment, and xenophagy (53, 54). We observed NADPH oxidase recruitment in TMZ-treated *Atg5* cKO macrophages, yet TMZ failed to restrict growth of Mtb in the absence of xenophagy, arguing against direct antimycobacterial activity of the NADPH oxidase, consistent with other recent studies (40, 55). We conclude that the antimycobacterial activity of ROS is primarily based upon its ability to activate lysosomal trafficking pathways or depends upon a combination of lysosomal trafficking and ROS.

The immunometabolic relationship between FAO, mROS, and macrophage effector functions was unanticipated and has important implications for studies outside innate immunity, as FAO inhibitors are proposed for metabolic therapy in cancer (56, 57) and heart failure (58), and mROS may contribute to their mechanism of action. While the current view is that ROS generation contributes to inflammation and cellular damage, a beneficial role of RET-ROS was appreciated recently for hypoxia sensing in the carotid body (59), ischemia-reperfusion injury (60), and extending fly life span (61). TMZ is an attractive compound for generating mROS, as it has been used to treat angina for 35 years, is orally bioavailable, lacks substantial drug-drug interactions, and has a favorable safety profile (62). In the context of antimicrobial therapy, HDTs would need to be used in combination with directly acting antimicrobials, where their addition might contribute to treatment-shortening regimens. While the *in vivo* effects of FAO inhibition were modest during Mtb infection, they occurred at drug concentrations that are used clinically and were on par with other host-directed approaches, such as those taking an autophagy-based strategy, which are currently being evaluated in clinical trials (21, 22, 63–65). The effect in mice will reflect that impact of impairing FAO in a variety of cell types, and addressing this complexity, as well as their activity in combination with directly acting antimicrobials, is an area for future investigation. The more important point of our work is that we report that the stimulatory effect of mROS can restore NADPH oxidase activity and LC3 trafficking during Mtb infection. Whether more substantial antimicrobial control could be established by a combination of HDTs that target distinct cellular reservoirs or work synergistically to activate antimicrobial pathways will also be critical to investigate.

## MATERIALS AND METHODS

### Reagents and antibodies.

FAO inhibitors used in the study were etomoxir (catalog no. 4539; Tocris Bioscience), trimetazidine (catalog no. 18165; Cayman Chemical Company), and oxfenicine (catalog no. 56160; Millipore Sigma). Metformin (catalog no. D150959), mitoTEMPO (catalog no. SML0737), diphenyleneiodonium chloride (DPI) (catalog no. D2926), *N*-acetyl cysteine (NAC) (catalog no. A7250), *tert*-butylhydroperoxide (tBHP) (catalog no. 416665), amikacin (catalog no. A1774), phorbol 12-myristate 13-acetate (PMA) (catalog no. P8139), rapamycin (catalog no. R0395), rotenone (catalog no. R8875), antimycin (catalog no. A8674), and fluorocarbonyl cyanide phenylhydrazone (FCCP) (catalog no. C2920) were obtained from Millipore Sigma. The fluorescent dyes CellRox green (catalog no. C10444), MitoSox red (catalog no. M36008), calceinAM (catalog no. C1430), BODIPY 493/503 (4,4-difluoro-1,3,5,7,8-pentamethyl-4-bora-3a,4a-diaza-s-indacene) (catalog no. D3922) were obtained from Thermo Fisher Scientific. For immunostaining, we used rabbit polyclonal primary antibodies against gp91^phox^ (ab80508; Abcam), p40^phox^ (sc-18252-R; Santa Cruz Biotechnology), and LC3B, mouse monoclonal antibody against p62 (catalog no. IC8028G; R&D Systems), and secondary antibody Alexa Fluor 488 goat anti-rabbit IgG (H+L) (catalog no. A11034; Thermo Fisher Scientific).

### Bacterial strains.

Mycobacterium tuberculosis (Mtb) and Mycobacterium abscessus were grown at 37°C to log phase in Middlebrook 7H9 broth (BD Biosciences) supplemented with 0.05% Tween 80, BD BBL Middlebrook ADC Enrichment (BD Biosciences), and 0.2% (vol/vol) glycerol. Plasmids were selected with 25 μg/ml kanamycin or 50 μg/ml hygromycin depending on the resistance marker. H37Rv, the wild-type Mtb strain, Δ*katG*, and Δ*esxA* mutant strains were provided by William Jacobs, Jr. (Albert Einstein College of Medicine) (54). dsRed-expressing H37Rv was a gift from J. Ernst (New York University). H37Rv expressing the Vibrio harveyi luciferase (Rv-lux) was a gift from Jeffery Cox (University of California, Berkeley). The “live-dead” reporter strain constitutively expresses mCherry and inducibly express green fluorescent protein (GFP) under the control of a tetracycline-inducible promoter (27).

### Mice.

We used 8- to 12-week-old C57BL/6 mice. *Cpt2^fl/fl^* LysM-Cre^+^ mice were generated by crossing *Cpt2^fl/fl^* and LysM-Cre^+^ mice. The generation of *Cpt2^fl/fl^* and LysM-Cre^+^ mice have been described previously (66, 67). The Washington University School of Medicine Institutional Animal Care and Use Committee approved all the work with mice. Euthanasia was performed prior to bone marrow harvest in accordance with the 2013 *AVMA Guidelines for the Euthanasia of Animals* (68).

### Cell culture.

To obtain murine bone marrow-derived macrophages (BMDMs), marrow was flushed from the femurs and tibia of mice, and the hematopoietic stem cells were allowed to differentiate for 7 days in Dulbecco’s modified Eagle medium (DMEM) (Gibco) supplemented with 10% fetal bovine serum (FBS) (Gibco), 1% penicillin-streptomycin (Pen-Strep) solution (Gibco), and 20% L929 conditioned medium. After 7 days, the BMDMs were harvested using Ca^2+^/Mg^2+^-free phosphate-buffered saline (PBS) (Gibco) containing 5 mM EDTA (Invitrogen, Life Technologies), and maintained in DMEM containing 10% FBS and 10% L929 conditioned medium after infection. Immortalized BMDMs (iBMDMs) were immortalized by infection with the J2 retrovirus (BEI Resources). RAW 264.7 and THP-1 cells were obtained from American Type Tissue Collection (ATCC) and were maintained in DMEM and RPMI 1640, respectively, with 10% FBS. THP-1 differentiation was induced using 20 ng/ml phorbol 12-myristate 13-acetate (PMA) (Sigma) for 18 to 20 h.

### Bacterial infections.

For *in vitro* macrophage assays, a log-phase culture of Mtb H37Rv was pelleted and resuspended in macrophage culture medium. Bacterial single-cell suspensions were prepared by filtering through 5-μm filters (catalog no. 4650; PALL Life Sciences). The number of Mtb in the resultant filtrate was estimated by measuring absorbance at 600 nm, followed by infection of macrophages at a multiplicity of infection (MOI) of 5. After 4 h, macrophages were washed three times with warm DMEM to remove extracellular bacteria, and then resuspended in medium containing FAO inhibitors or solvent control. To estimate intracellular Mtb growth, infected macrophages were lysed in 0.06% sodium dodecyl sulfate (SDS) solution at the indicated time points, and serial dilutions of the lysates were plated on 7H11 agar plates (catalog no. 283810; BD Biosciences) containing BD BBL Middlebrook OADC (oleic acid-albumin-dextrose-catalase) enrichment (catalog no. 212351; BD Biosciences) and glycerol. The numbers of CFU were calculated 14 to 21 days later. For *in vivo* infections, log-phase H37Rv culture was pelleted and resuspended in sterile 0.5% Tween 80 solution. After a centrifugation step, the supernatant was used for aerosol infection using a Glas-Col inhalation exposure system. The infectious dose administered was calculated by plating CFU from an aliquot of the bacterial suspension.

### Mouse surgeries and aerosol infections.

Alzet mini-osmotic pumps (model 2002; Durect Corporation, CA) were loaded with saline or TMZ solution as per the manufacturer’s protocol. The osmotic pumps were surgically implanted in anesthetized mice under aseptic conditions. The mice were administered analgesic to minimize pain and were monitored regularly for signs of pain and other postoperative complications. Two days later, the mice were infected with Mtb H37Rv via aerosol route using an inhalation exposure system from Glas-Col. The dose of infection was confirmed 1 day postinfection by plating whole-lung homogenates from two mice on Middlebrook 7H11 agar. Two weeks postinfection, the mice were euthanized, and the lungs were harvested, homogenized, and plated for CFU.

### Serum microsampling of TMZ.

Serum concentrations of TMZ in mice were determined at 5 and 14 days after initiating treatment. Lidocaine was applied to mouse tails to minimize pain, and the end of the tail was wiped with alcohol, and a small incision was made. One hundred microliters of blood was collected in K_2_-EDTA microvette tubes (Braintree Scientific, Inc.) and was centrifuged at 5,000 rpm for 5 min to recover plasma. Samples were stored at – 80°C until analyzed for TMZ content.

### Fluorescence microscopy and image analyses.

BMDMs were seeded in 8-well chamber slides (Falcon culture slide 8-well, catalog no. 08-774-26), and infected with dsRed-expressing H37Rv at an MOI of 5. At the indicated time points, samples were fixed overnight with 1% paraformaldehyde (PFA). For immunofluorescence (IF), samples were permeabilized and blocked in PBS with 0.05% saponin and 3% bovine serum albumin (BSA), and stained with the indicated primary antibodies for 2 h at room temperature or overnight at 4°C. Primary antibodies used were p40^phox^, gp91^phox^/NOX2, LC3, and p62. Staining with Alexa fluorophore-conjugated secondary antibody was done for 2 h at room temperature. Following this, the samples were washed with 0.1% Tween 20/PBS and mounted using Prolong Gold antifade (catalog no. P36930; Thermo Fisher Scientific).

Images were captured using a Nikon Eclipse Ti confocal microscope (Nikon Instruments Inc.) equipped with a 60× apochromat oil-objective lens, and analyzed using NIS-Elements version 4.40 (Nikon). Briefly, a region of interest (ROI) was drawn around each bacterium, and the mean fluorescence intensity (MFI) was measured using the ROI statistics tool.

### ROS measurement assays.

For ROS measurement assays, the macrophages were seeded in 96-well plates (μ-Plate 96 well [catalog no. 89626; IBIDI]). To estimate total cell ROS, the samples were treated with CellRox green fluorescent dye (Thermo Fisher Scientific) at 5 μM for 30 min. The samples were washed three times with PBS and fixed overnight with 1% PFA. Mitochondrial ROS was measured in live, uninfected macrophages using MitoSox fluorescent dye (Thermo Fisher Scientific) at 5 μM for 30 min. The samples were imaged using confocal microscopy, and the ROS signal of each cell was quantified using NIS-Elements software. Briefly, each cell was converted to a ROI, and the MFI was measured using the ROI statistics tool.

### Oxygen consumption rate and extracellular acidification rate.

BMDMs from *Cpt2* cKO mice and control littermates were plated in 96-well Seahorse plates at a density of 75,000 cells per well. The cells were treated with 5 nM TMZ or solvent control for 3 h. After treatment, the cells were washed and placed in XF medium (nonbuffered RPMI 1640 containing 25 mM glucose, 2 mM l-glutamine, and 1 mM sodium pyruvate) with 10% fetal calf serum (FCS). Oxygen consumption rate (OCR) and extracellular acidification rates (ECARs) were measured under basal conditions, and the following inhibitors were added: 1 μM oligomycin (Sigma), 1.5 μM fluorocarbonyl cyanide phenylhydrazone (FCCP) (Sigma), and 100 nM rotenone (Sigma) plus 1 μM antimycin A (Sigma). Measurements were taken, using a 96-well extracellular flux analyzer (Seahorse Bioscience, North Billerica, MA, USA).

### Cytokine measurements.

Culture supernatants were harvested from uninfected or Mtb-infected BMDMs that were untreated or treated with 5 or 50 nM TMZ for 24 and 72 h. The conditioned medium was filter sterilized, and cytokines and chemokines were measured using Milliplex MAP Mouse Cytokine/Chemokine Magnetic Bead Panel (MCYTOMAG-70K;Millipore Sigma) and Procartaplex Mouse IFNα/IFNβ Panel (2plex) (Thermo Fisher Scientific).

### Liquid MIC determinations.

In 96-well plates, Mtb cultures with a starting optical density at 600 nm (OD_600_) of 0.01, 0.05, and 0.10 were incubated with increasing concentrations of drugs in triplicate. The growth of Mtb was measured at days 0, 1, 2, 3, and 4. The MIC was considered the minimal concentration tested that inhibited Mtb growth at 4 days. To assess the direct toxicity of TMZ on Mtb, H37Rv expressing the Vibrio harveyi luciferase (Rv-lux) was cultured in Middlebrook 7H9 broth with or without 1 mM TMZ for 7 days. Relative luminescence units (RLU) were measured every 2 days at 490 nm.

### PK studies.

Pharmacokinetic (PK) and plasma binding studies were performed by Alliance Pharma (PA, USA) following intravenous (IV) and oral administration of 3 and 15 mg of TMZ/kg of body weight, respectively. PK study of TMZ using Alzet osmotic pumps was conducted by Paraza Pharma Inc., Canada. Briefly, Alzet osmotic pumps (model 2002; Durect Corporation) were surgically implanted in C57BL/6 mice (*n* = 5) for a subcutaneous infusion at 10.66 mg/kg/day. Plasma TMZ concentrations were determined over the course of 48 h.

### Statistics.

GraphPad Prism software was used to prepare plots and assess statistical significance of results using unpaired Student's *t* test with Welch’s correction, ordinary one-way analysis of variance (ANOVA) and Mann-Whitney test.
