# Supplementary figures and images for: Inhibition of Fatty Acid Oxidation Promotes Macrophage Control of Mycobacterium tuberculosis
Source: mBio. 2020 Jul 7;11(4):e01139-20. doi: 10.1128/mBio.01139-20 (PMC7343992; doi:10.1128/mBio.01139-20)

**Fig. S1**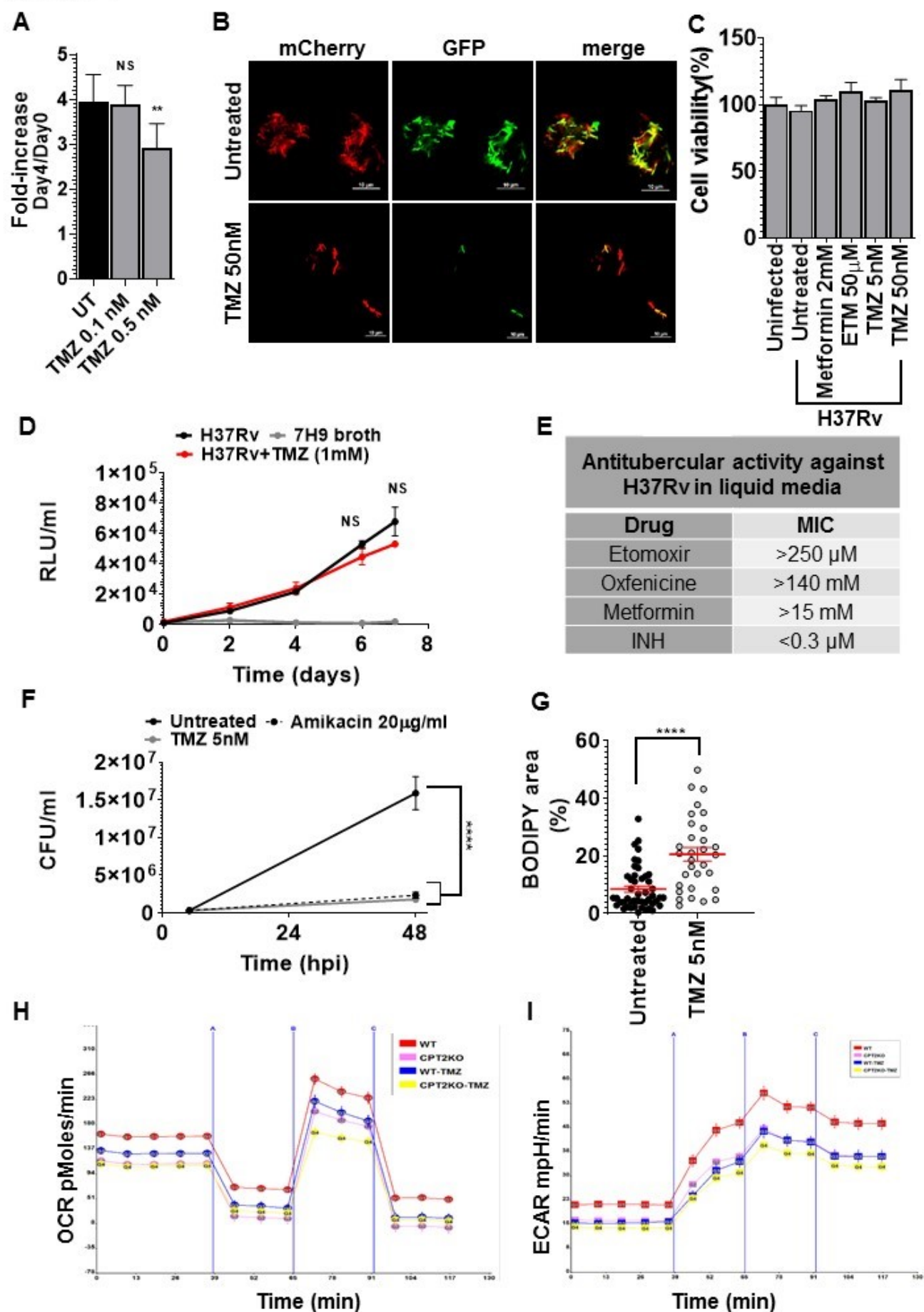

Supplement: FIG S1 [file mBio.01139-20-sf001.pdf]

# Fig. S3

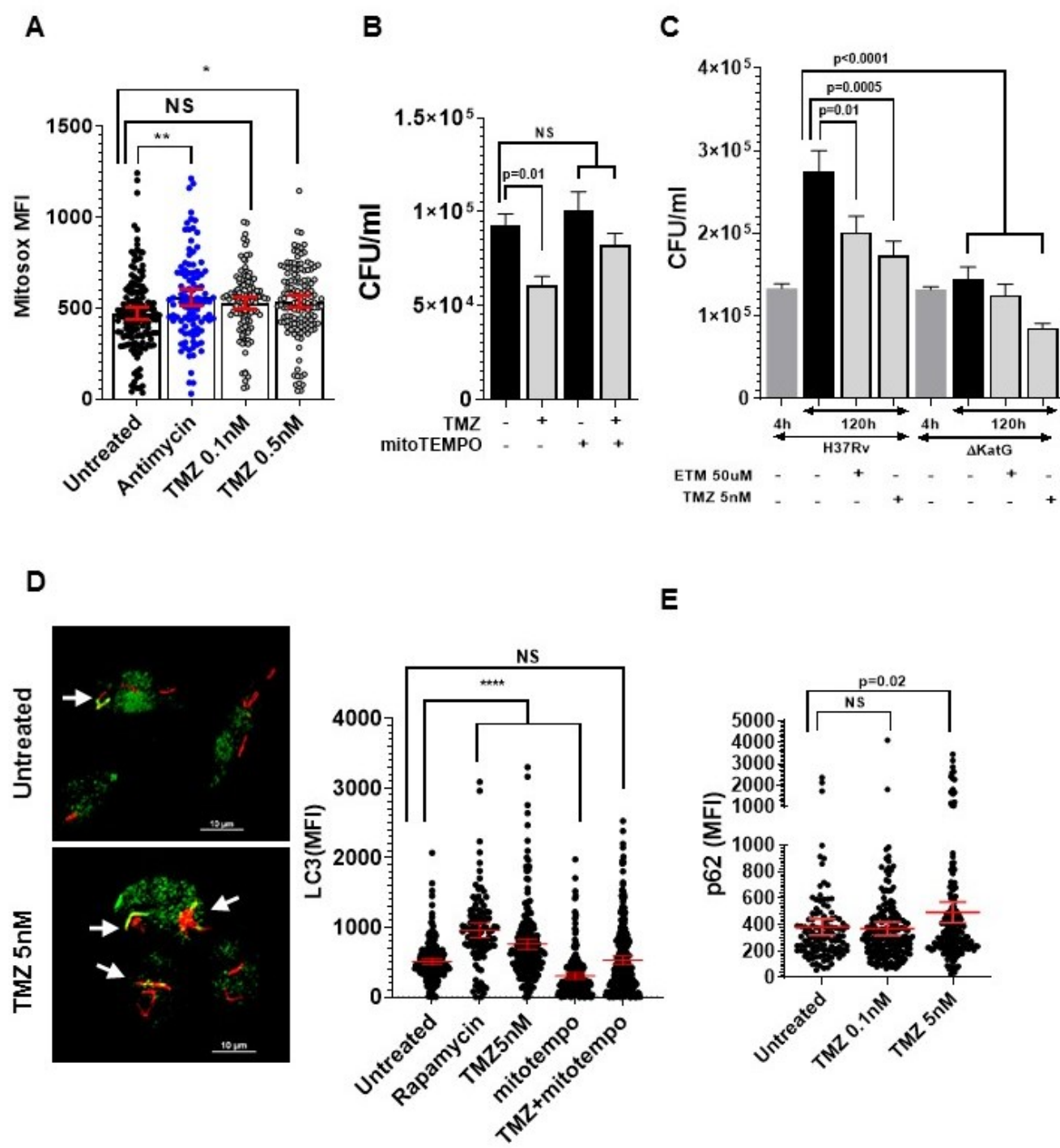

Supplement: FIG S3 [file mBio.01139-20-sf003.pdf]

**Fig. S4**

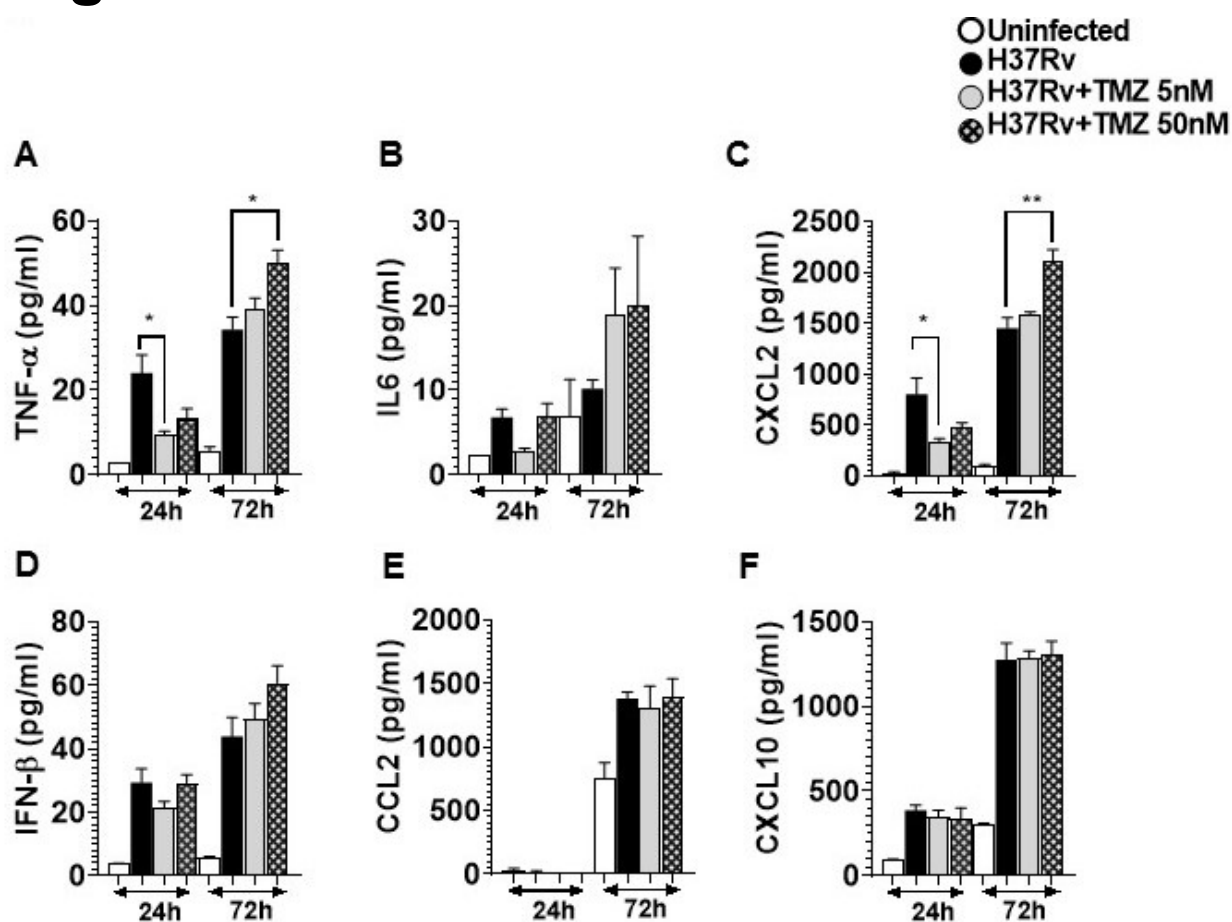

Supplement: FIG S4 [file mBio.01139-20-sf004.pdf]
