# Supplementary material for: Inhibition of Fatty Acid Oxidation Promotes Macrophage Control of Mycobacterium tuberculosis
Source: mBio. 2020 Jul 7;11(4):e01139-20. doi: 10.1128/mBio.01139-20 (PMC7343992; doi:10.1128/mBio.01139-20)

Fig. S2

A

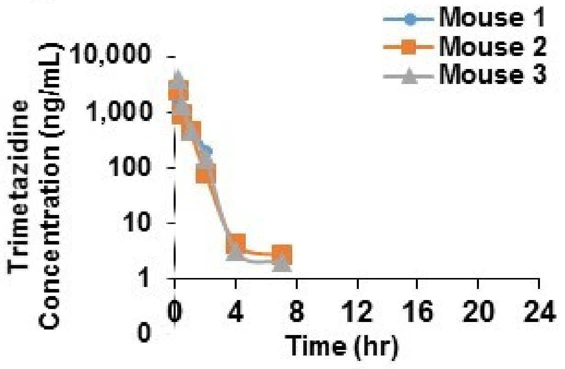

B

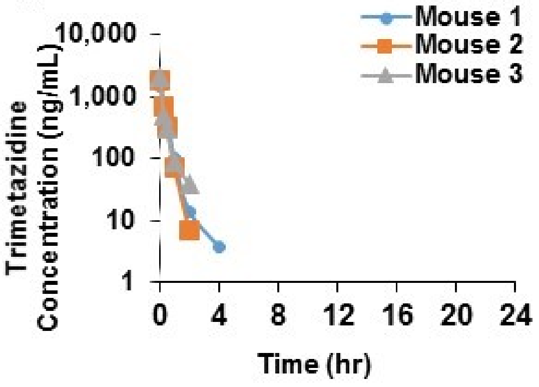

C

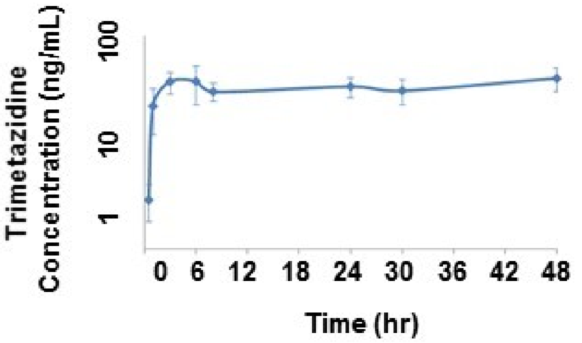

D

Protein Binding in Mouse Plasma		
Compound	Percent Free Fraction	Percent Recovery
Trimetazidine	64.86	97.94
Warfarin	4.55	110.55

E

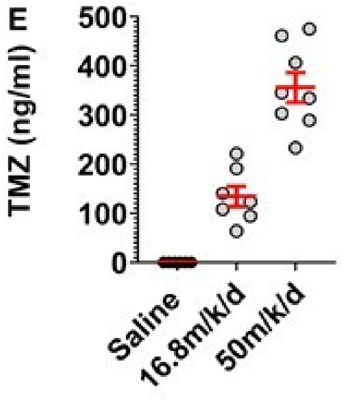

F

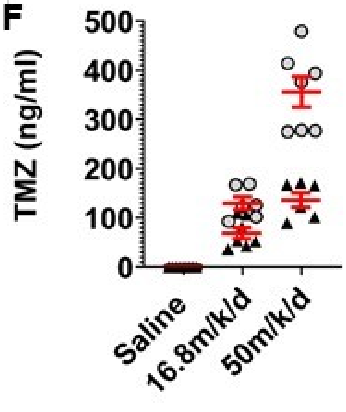

Supplement: FIG S2 [file mBio.01139-20-sf002.pdf]
